# High school dropout and long-term sickness and disability in young adulthood: a prospective propensity score stratified cohort study (the Young-HUNT study)

**DOI:** 10.1186/1471-2458-13-941

**Published:** 2013-10-09

**Authors:** Karin A A De Ridder, Kristine Pape, Koenraad Cuypers, Roar Johnsen, Turid Lingaas Holmen, Steinar Westin, Johan Håkon Bjørngaard

**Affiliations:** 1 Department of Public Health and General Practice, Norwegian University of Science and Technology, 7491 Trondheim, Norway; 2 Youth Health Care, Department of Public Health and Primary Care, KU Leuven, 3000 Leuven, Belgium; 3 Department of Public Health and Surveillance, Scientific Institute of Public Health, WIV-ISP (Site Elsene), 1050 Brussels, Belgium; 4 Department of Public Health and General Practice, HUNT Research Center, Norwegian University of Science and Technology, 7600 Levanger, Norway; 5 Forensic Department and Research Centre Bröset, St. Olav's University Hospital Trondheim, 7491 Trondheim, Norway

**Keywords:** School dropouts, Sick leave, Disability insurance, Risk factors, Adolescent, Adult, Prospective cohort study, Propensity score, HUNT study

## Abstract

**Background:**

High school dropout and long-term sickness absence/disability pension in young adulthood are strongly associated. We investigated whether common risk factors in adolescence may confound this association.

**Methods:**

Data from 6612 school-attending adolescents (13–20 years old) participating in the Norwegian Young-HUNT1 Survey (1995–1997) was linked to long-term sickness absence or disability pension from age 24–29 years old, recorded in the Norwegian Labour and Welfare Organisation registers (1998–2008). We used logistic regression to estimate risk differences of sickness or disability for school dropouts versus completers, adjusting for health, health-related behaviours, psychosocial factors, school problems, and parental socioeconomic position. In addition, we stratified the regression models of sickness and disability following dropout across the quintiles of the propensity score for high school dropout.

**Results:**

The crude absolute risk difference for long-term sickness or disability for a school dropout compared to a completer was 0.21% or 21% points (95% confidence interval (CI), 17 to 24). The adjusted risk difference was reduced to 15% points (95% CI, 12 to 19). Overall, high school dropout increased the risk for sickness or disability regardless of the risk factor level present for high school dropout.

**Conclusion:**

High school dropouts have a strongly increased risk for sickness and disability in young adulthood across all quintiles of the propensity score for dropout, i.e. independent of own health, family and socioeconomic factors in adolescence. These findings reveal the importance of early prevention of dropout where possible, combined with increased attention to labour market integration and targeted support for those who fail to complete school.

## Background

Young people dropping out from school, never being included in or leaving the labour market due to health problems or disability represent an individual hazard and a society challenge [1,2]. Prospective studies of health and social functioning in young adulthood among dropouts are rare, although there is evidence to suggest a substantially higher risk of sickness and disability among high school dropouts compared to school completers [3,4]. Hence, a better understanding of the complex role of adolescent health and socioeconomic factors underlying the association between school dropout and subsequent sickness and disability may provide important information for social welfare strategies and for public health policy.

The association between school dropout and subsequent sickness and disability could be confounded by the co-occurrence of lower childhood socioeconomic position (SEP), adolescent ill health and other risk factors [5-16]. In a life-course framework, the accumulation of risks may be clustered and often be related to the family’s socioeconomic position in society [17]. Hence, baseline differences in risk profiles between high school dropouts and completers, to a large extent, may explain their further trajectories in adulthood and their risk for long-term sickness and disability [18,19]. Another life-course framework model is the chain of risk model, which resembles what has been described as a “pathways model” [17], where each exposure increases the risk of a subsequent exposure, but in addition to an independent effect on the outcome irrespective of the later exposure.

In a large prospective study of about 6612 Norwegians, we investigated the role of adolescent health, health-related behaviours, psychosocial factors, school problems and parental socioeconomic position in the association between high school dropout and long-term sickness absence or disability pension in young adulthood. We hypothesized that the more vulnerable adolescents with a high risk level for school dropout would, in case of school dropout, have an even greater increased risk for long-term sickness absence or disability pension compared to the adolescents with a low risk level for school dropout.

## Methods

### Participants

Young-HUNT is the adolescent part of the HUNT Study (The Nord-Trøndelag Health Study, http://www.ntnu.no/hunt) in the county of Nord-Trøndelag, Norway [20]. All school attending students of middle and secondary school in 1995–97 were invited to participate in the Young-HUNT1 Survey, and 8949 adolescents (90% response rate) completed a comprehensive questionnaire during a class hour. Data from Young-HUNT1 were linked to information about social insurance benefits from the Norwegian Labour and Welfare Organisation registers (FD-trygd) in the period 1998–2008. Adolescents and their parents were linked to the Norwegian National Education Database (http://www.ssb.no/mikrodata). Parents and siblings (those with the same biological mother) were identified through the national identity number in the Norwegian national family register.

We excluded 2333 adolescents from this study. Causes for exclusion were disability pension collected within the period (16–21 years old) when they were eligible for high school education (30), missing educational data (8), death before age 24 (30), migration before age 24 (57), born after 1983 (4) or age-school level mismatch (4). Because of complete cases analyses, 2204 individuals were excluded due to missing data on the questionnaire or the physical examination (BMI).

The present study was approved by The Regional Committee for Medical Research Ethics (reference 2010/1527-5), and was conducted according the Declaration of Helsinki. Each participant and the parents/legal guardians of the participants younger than 16 years old gave their written consent to participate in the Young-HUNT Study.

### Long-term sickness absence or disability pension

The outcome was long-term sickness absence or disability pension defined as medical benefits for permanent and temporary disability pension, medical, and vocational rehabilitation or sickness benefits received at least 180 days in one calendar year. This was based on annual registrations from the National Insurance Administration in the period 1998 to 2008 and defined as at least one episode of long-term medical benefits in a calendar year during the six-year follow-up period between age 24 and 29 years.

### School dropout

Basic education in Norway is compulsory up to the start of senior high school (upper secondary education) at age 16. Every 15- to 16-year-old has a statutory right to 3 years of senior high school which consists of both general and vocational tracks. In the follow-up period (1998–2008), we registered the outcome high school for all participants as either having obtained (completion) or having *not* obtained (dropout) a certificate of senior high school (general or vocational track) in the calendar year the participant turned 24 years old. We chose to measure dropout at a later point estimate to avoid overestimation of the dropout rates because of the flexibility in study options and to make international comparison easier, because it is less dependent of the national school structure [2]. Data were retrieved through linkage to the Norwegian National Education Database which coded level of education by NUS2000-standards, which implemented the international education standard ISCED97.

### Covariates

We defined the characteristics of the participants according to demographic data (age and sex), follow-up time, health, health behavior, psychosocial factors, school-related factors, and maternal education level. Follow-up time was the number of years from age 24 to end of follow-up or maximum age 29 in the period 1998–2008 when alive or not migrated. Maternal education level was registered at the time the participant was 16 years old and divided into three categories: compulsory (primary and lower secondary education), intermediate (upper secondary and post-secondary non-tertiary education) and tertiary (under-graduate, graduate and post-graduate education). Assessments of health and health behavior were based on the self-reported information from the participants in the Young-HUNT1 Survey (1995–1997): somatic disease (asthma, diabetes, migraine, epilepsy, or other longstanding illness), somatic symptom load, psychological distress, concentration difficulties, insomnia, self-rated health, smoking, and physical activity level. Trained nurses measured height and weight following a standard protocol. Body mass index (BMI) was defined by cutoffs for the appropriate age groups as proposed by Cole et al. [21]. Psychosocial factors included self-esteem, subjective well-being, loneliness, and family living situation. School-related factors included self-reported reading and writing difficulties, bullying, disease-related school absence, educational aspirations, academic problems, school dissatisfaction, and school-related conduct. (see Additional file 1: Table A for operational definition of the covariates).

### Statistical methods

We presented baseline characteristics of participants who completed or dropped out of high school. Primary analysis investigated the association between high school dropout and long-term sickness or disability between ages 24 and 29. We used sex-, age- and follow-up time adjusted logistic regression on complete datasets (N=6651). Logistic regression was preferred above Cox regression analyses because we were mainly interested in estimating the *absolute* risk difference (and the effect of known confounders on this risk difference), rather than assessing the relative risk of receiving benefits for a person at risk per unit time. To adjust for possible confounders, we successively added maternal education level, health measures, health behavior, psychosocial factors, and school-related factors*.* We carried out tests for statistical interaction between high school dropout and sex and between high school dropout and maternal education level. Since a quarter of the study population had missing data at baseline, we also performed a sensitivity analysis with multiple imputations by chained equations (MICE) procedures to obtain 20 imputed datasets, which included most of the participants who had missing data (N=8805) (see Additional file 1: Table C for details about the imputation modeling procedure) [22]. Using the rich information in the Young-HUNT study to impute missing data, we assumed that missing data were missing at random. Many variables that are associated with non-participation in surveys were included in the dataset, which reduces the probability that data missing does depend on unobserved data, conditional on the observed data (see Additional file 1: Table B for description of missing data). The multiple imputation analyses are not presented as the main analyses as it was technically impossible to perform an imputation without comprehensive manipulation of the data, such as redefinition of the continuous variables into binary or ordinary variables and exclusion of the variable “academic problems” (important to calculate the propensity score) because of collinearity.

We also estimated multivariable conditional logistic regression models in order to control for factors that are shared within families (Number of siblings=316). By conditioning on the family of origin, these models compare long-term sickness or disability among sibships with and without high school dropout while controlling for all family background characteristics (observed and unobserved) that the siblings share [23]. These models were adjusted for sex, age, and follow-up time. Successively, we added health measures, health behavior, social factors, and school-related factors.

To investigate conditional vulnerability of dropout, we computed the propensity score (from 0 to 1) by using logistic regression; the dependent variable was high school dropout and the independent variables (covariates) were sex, age, maternal education level, health and health behavior measures, psychosocial factors, and school-related factors. The propensity score is a calculation of the probability to drop out of high school for a participant with specific predictive factors (regardless of whether they dropped out of high school or not). We computed the quintiles of the estimated propensity score with the first quintile representing the lowest probability to drop out of high school and the fifth quintile representing the highest probability. Within these strata, the covariates in the groups with high school dropout and completers are similarly distributed [24]. We carried out a logistic regression analysis with a statistical interaction between high school dropout and the propensity score stratified by quintiles.

As a sensitivity analysis, we also obtained a weighted estimate of the pooled odds ratio across the propensity score strata. Furthermore, we used propensity score matched methods in STATA to estimate the average treatment effect on the treated (ATT), or in our case “the average dropout effect on the dropouts”, based on the propensity score. We used the technique radius matching with a propensity score radius of 0.1 [25].

Data were analyzed with STATA 12.1 (StataCorp LP). Odds ratios (OR) and risk differences (RD) were presented with 95% confidence intervals (CI). Risk differences were estimated from the logistic regression analyses with the covariates at their mean and follow-up time (from age 24 to 29) at 6 years.

## Results

The study cohort with complete datasets (N=6612) consisted of 3375 girls (51%) and 3237 boys (49%). The baseline mean age of the participants was 16.1 years old (range 13 to 20 years). The mean follow-up time from age 24 to 29 was 4.5 years (range 1 to 6 years). During the follow-up period between the ages 24 and 29, 739 (11%) had long-term sickness or disability, more girls (13%) than boys (9%).

Overall, at the age of 24, 910 (14%) had not completed high school. High school dropouts were more likely than completers to be male, to have a mother with low education and less likely to live in a traditional family. In addition, they were more likely to have health problems, to smoke, to be physically inactive, to be lonely or bullied, and to have reported lower self-esteem and school related problems (Table 1).

**Table 1:** Baseline characteristics of high school dropouts and high school completers (N= 6612)

	**School dropouts (n=910)**		**School completers (n=5702)**	
*Demographics*				
Age, mean, yr	16.09	(15.86-16.11)	16.10	(16.04-16.14)
Male	58.57	(55.36-61.77)	47.42	(46.13-49.72)
*Maternal education level*				
Primary	41.09	(37.90-44.30)	23.41	(22.31-24.51)
Intermediate	47.14	(43.90-50.39)	50.84	(49.54-52.14)
Tertiary	11.79	(10.68-13.85)	25.75	(24.61-26.88)
*Health*				
1 or more somatic disease	24.73	(21.92-27.53)	19.66	(18.63-20.69)
Symptom load, mean	1.60	(1.49-1.70)	1.31	(1.26-1.35)
High psychological distress, mean	1.52	(1.48-1.56)	1.45	(1.43-1.46)
Concentration problems	36.59	(33.46-39.72)	21.76	(20.70-22.84)
Insomnia	14.07	(11.81-16.33)	9.53	(8.76-10.28)
Poor self-rated health	17.14	(14.69-19.59)	9.27	(8.52-10.03)
*Health behavior*				
BMI				
Overweight	18.46	(15.94-20.98)	13.47	(12.58-14.36)
Obese	5.49	(4.01-6.98)	2.59	(2.18-3.01)
Smoking	34.62	(31.52-37.71)	18.82	(17.80-19.83)
No physical activity	19.67	(17.09-22.25)	10.93	(10.12-11.74)
*Psychosocial factors*				
Self-esteem, mean	2.95	(2.91-2.98)	3.05	(3.03-3.06)
Subjective well-being, mean	2.86	(2.80-2.93)	2.68	(2.65-2.70)
Loneliness, mean	2.10	(2.03-2.16)	2.01	(1.97-2.03)
Traditional family	59.12	(55.92-62.32)	77.50	(76.41-78.58)
*School-related factors*				
Reading and writing difficulties	15.60	(13.24-17.96)	6.42	(5.78-7.05)
Being bullied, mean	1.23	(1.19-1.26)	1.16	(1.15-1.17)
Disease-related school absence	9.67	(7.75-11.59)	3.95	(3.44-4.45)
Aspiration for higher education	38.57	(35.41-41.91)	46.62	(45.32-47.91)
Academic problems, mean	2.15	(2.12-2.18)	1.87	(1.86-1.89)
School-related dissatisfaction, mean	2.40	(2.36-2.44)	2.26	(2.25-2.28)
School-related conduct, mean	1.59	(1.56-1.62)	1.45	(1.44-1.46)

The numbers are proportions (in %), unless stated otherwise, with 95% confidence intervals between parentheses.

The regression analyses displayed in Table 2 show the associations between high school dropout and long-term sickness or disability between ages 24 and 29. In the crude model, the risk difference for long-term sickness or disability for high school dropouts compared with high school completers was 0.21 or 21% points (95% CI 17 to 25). With the successive adjustment for maternal education level, health measures, health behavior, psychosocial factors, and school-related factors, the risk difference gradually decreased to 15% points (95% CI, 12 to 19). There was no evidence for effect measure modification by sex or maternal education level (p-value for interactions > 0.1). The magnitude and direction of the differences in long-term sickness or disability in young adulthood based on the main analyses of complete data and the sensitivity analysis of multiple imputations were in accordance to those presented in Table 2 (see Additional file 1: Table C).

**Table 2:** Risk difference^* ^**and odds ratio with 95% confidence intervals for long-term sickness or disability between age 24 to 29 years for high school dropouts versus school completers in the whole population and within the families**

	**Model 0**	**Model 1**	**Model 2**	**Model 3**	**Model 4**	**Model 5**
**Dropout *versus *** **completion (ref.)**						
**Whole population**						
Risk difference	20.8 (17.0 to 24.7)	18.7 (15.0 to 22.4)	17.4 (13.8 to 21.1)	16.6 (13.0 to 20.4)	15.8 (12.2 to 19.5)	15.3 (11.7 to 19.0)
Odds ratio	3.92 (3.28 to 4.68)	3.53 (2.95 to 4.24)	3.34 (2.8 to 4.0)	3.20 (2.65 to 3.86)	3.07 (2.54 to 3.71)	2.96 (2.44 to 3.60)
**Within family**^**1**^						
Odds ratio	1.89 (0.96 to 3.74)	–	2.03 (1.01 to 4.08)	2.53 (1.15 to 5.54)	2.48 (1.13 to 5.49)	2.39 (1.04 to 5.47)

*Estimated risk difference in the 6-year risk for long-term sickness and disability with the covariates at their mean.

Risk difference (in %, with 95% CI) and odds ratio (with 95% CI) in the whole population (logistic regression models, N=6612) and within the families (sibling fixed-effect models, N=316).

Model 0: adjusted for sex, age, and follow-up time.

Model 1: model 0 +adjusted for maternal education level.

Model 2: model 1 + adjusted for somatic disease, symptom load, psychological distress, concentration problems, insomnia, and self-rated health.

Model 3: model 2 + adjusted for overweight, smoking, and physical activity.

Model 4: model 3 + adjusted for self-esteem, subjective well-being, loneliness, and family living situation.

Model 5: model 4 + adjusted for reading and writing difficulties, bullying, disease-related school absence, educational aspirations, academic problems, school dissatisfaction, and school-related conduct.

^1^In the Within-family models the covariate maternal education level is omitted.

**Figure 1:**
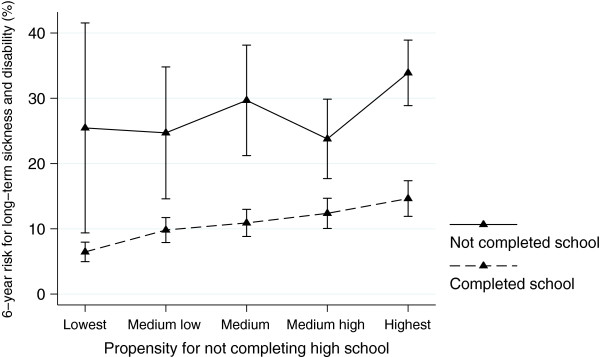
Estimated 6-year risk for long-term sickness or disability with 95% confidence intervals at age 24–29 according to the adolescents’ propensity for not completing high school and high school graduation status (N=6612).

The sibling analysis confirmed the results from the total population, but the odds ratios were substantially lower (Table 2). The precision was reduced due to reduced statistical power in the within-family models. Table D (see Additional file 1) presents the variables that were included in the propensity score analysis, along with the regression coefficients and standard errors. The c-index for the propensity score was 0.76, and figure A (see Additional file 1) visualizes the overlap between the two groups (high school dropouts and completers) on the propensity score. Table 3 presents the risk differences and odds ratios for long-term sickness or disability for high school dropouts compared to high school completers for each stratum of the propensity score. Overall, a high school dropout had a higher risk for long-term sickness or disability in each stratum. The pooled odds ratio across the propensity score strata was 2.95 (95% CI, 2.44 to 3.57), which results in an estimated risk difference between school dropouts and completers of 16.7% points (95% CI, 12.2 to 21.3). This is similar to the estimated ATT of 0.165 (95% CI, 0.136 to 0.194) in the radius matched propensity score analyses (see Additional file 1: Table E). A high school *completer* in stratum 1 (lowest risk) had a 7% (95% CI, 5 to 8) risk for long-term sickness or disability, while a high school *dropout* in stratum 5 (highest risk) had a 34% (95% CI, 29 to 39) risk (Figure 1). Compared to a participant in stratum 1, a person in stratum 5 had 7% points (95% CI, 4 to 10) higher risk for long-term sickness and disability. We found weak evidence of effect measure modification between the propensity score and dropout (p-value for interaction > 0.1).

**Table 3:** Risk difference and odds ratios for long-term sickness or disability with 95% confidence intervals between the ages 24 and 29 years for school dropouts compared with school completers within each stratum of propensity score for dropping out of high school (N=6612)

	**N Medical benefits**	** RD (CI)**	** OR (CI)**
Lowest propensity	79	18.1 (2.3 to 33.9)	4.76 (2.00 to 11.37)
Medium low	120	16.4 (5.8 to 27.1)	3.16 (1.78 to 5.63)
Medium propensity	142	18.7 (10.3 to 27.2)	3.43 (2.20 to 5.33)
Medium high	159	11.0 (4.7 to 17.3)	2.20 (1.50 to 3.23)
Highest propensity	239	19.5 (14.1 to 24.8)	3.11 (2.31 to 4.18)

Risk difference (RD, in %, with 95% CI) and odds ratios (OR, with 95% CI).

## Discussion

In this large prospective study, we found a strong association between high school dropout and long-term sickness or disability in young adulthood even after adjustment for parental socioeconomic position, health in adolescence, health-related risk behaviours, psychosocial risk factors, and school problems. Not only did a high school dropout systematically have a higher risk for long-term sickness and disability independent of propensity to drop out, but also a high school completer with the highest predicted tendency to drop out (high risk factor level present) had a lower risk for medical benefits than a school dropout with the lowest predicted tendency to dropout (low risk factor level present).

### Strengths and limitations

The strengths of the study are the high number of participants, the prospective longitudinal design stratified by propensity score, and the robust associations. The main exposures (high school dropout and parental SEP) and outcome were based on nearly complete and high-quality national registers. The study population was school attending adolescents, and there was a high participation rate (90%). There might be more school dropouts among the non-responders and this might have led to some underestimation of the examined associations. The risk factors in adolescence relied on a self-reported questionnaire with missing data for a quarter of our study population, which might have caused bias; however sensitivity analyses with multiple imputed data produced comparable results. The number of sibling groups with different outcome status was low, and therefore these results, from the sibling comparison, should be interpreted with care. Because we measured the risk factors in adolescence only once at baseline, there could be some residual confounding. It is however unlikely that this could explain the strong association that remained after full adjustment. Other variables on personal characteristics, like self-regulation, coping behaviour, or intellectual performance, or on general interpretations, like social capital or social cohesion, might have been relevant.

### Previous literature

A few previous studies have investigated potential explanatory factors in adolescence for the association between educational level in general and long-term sickness or disability [4,19,26]. A Norwegian population based study found a higher risk for disability pension for high school dropouts when adjusted for parental position, low birth weight, and childhood disease benefits [4]. Two Scandinavian studies suggested that both educational level and IQ independently were associated with the risk of receiving disability pension [19,26]. We also found that the association between high school dropout and long-term sickness or disability pension remained strong, even when controlling for a larger variety of adolescent characteristics than in previous studies.

The associations between high school dropout and long-term sickness or disability attenuated, but remained strong when controlling for characteristics shared by the family. A Swedish twin study indicated that the association between educational level and disability pension could be attributed to childhood factors and genetic make-up [27]. However, they combined high school dropouts and completers in the same educational group, although dropouts have substantially higher risks than completers [3,4,19]. Nevertheless, some familial confounding might play an important role in understanding the causes of long-term medical benefits, and we might not have captured all the necessary characteristics related to the family, such as coping behaviours, familial health, and genetics [28-30].

Finally, we are not aware of any study which examines the risk of long-term sickness and disability considering the propensity to drop out of high school based on known risk factors and actual high school graduation status.

### Possible interpretations

A high school dropout had systematically a substantial higher risk for long-term sickness and disability, independent of the disadvantage or risk level for dropout that was observed in adolescence. Young adulthood is a stage of the life cycle were people acquire social roles, such as the work role, and school dropout is the first formal registration of own SEP and one’s future opportunities in the labour market. Whatever life course history, a school dropout is confronted with reduced work prospects and higher risk for increased job strain, more physical demands, lower self-esteem, and lower sense of coherence [31]. According to the present study’s results, the risk of health related exclusion following high school dropout cannot simply be identified by health-related behaviours, parental socioeconomic position, or other risk factors in adolescence. In a life-course approach study, low decision latitude as a young adult was strongly associated with later long term sickness absence, but the effect disappeared when educational attainment and childhood IQ were included in the analyses [32]. One possibility is that school dropouts face an increased risk in a “no exit” situation and are forced into social circumstances that offer no alternative choices. It might also be that they are less able to adapt successfully when they become ill because they lack qualifications and skills which their peers might develop at school or which are necessarily to maintain schooling. For a successful learning process, not only cognitive ability is important. Self-regulation has been shown the most essential asset for the willing to exert considerable effort to learn [33]. In the self-regulation construct, goal level, persistence, effort, and self-efficacy had the strongest effect on learning. Additionally, they might perceive their ability to change their environment and themselves in this environment differently. Personality and coping strategies might affect this perception, and subsequent schooling and labour market integration [34,35]. Finally, in the presence of ill health, there might be an increased risk for medicalization during the social process of school dropout and the possible subsequent reduced work integration, as with job loss and unemployment [36].

Our multivariable adjustments could explain about a quarter of the strong association in the adjusted analyses. Additionally, those with a high propensity to dropout had a higher risk for sickness and disability independent of completing high school or not, which may support the chain of risk model with additive effects [17]. Also the siblings fixed effect analyses showed that there might be some “general susceptibility” related to shared familial factors. Nevertheless, the robust and strong association that remained in all analyses suggests that the mechanisms involved in school dropout and young people’s subsequent integration in the labour market should be investigated and focused on in preventive strategies.

### Implications

High school dropout is a major public health challenge because it concerns many young people who are in danger of marginalization and social exclusion. Avoiding the main cause and preventing dropout based on a multidisciplinary approach so that children with disadvantages may succeed, should be a public health priority. However, it may be unrealistic to believe that a high school degree is obtainable by everybody. Nonetheless, there should be greater effort towards better integration in high school and in the labour market, including alternative school tracks in cooperation with the labour market and on the job competence-enhancing possibilities. Preferably, these should not be merely B-tracks, but socially accepted and valued alternatives based on learning by doing for those who strive to complete high school.

## Conclusions

Even for those born into and raised with good prospects, high school dropout strongly contributes to a problematic or failing of work integration due to impaired health. Future research and preventive measures should pay attention to school and work integration beyond the individual perspective, and include contextual factors in schools and families. It will demand a collaboration of school policies, labour market, public health policies, and research to find sustainable and socially accepted and valued alternatives.

## Abbreviations

BMI: Body mass index; MICE: Multiple imputation by chained equations procedures; SEP: Socioeconomic position; ATT: Average treatment effect on the treated.

## Competing interests

The authors declare that they have no competing interests.

## Authors’ contributions

All authors were involved in the design, contributed to the interpretation of the results, and approved the final version of the article. TLH is PI of the Young-HUNT Study and has been responsible for the Young-HUNT data collection. KDR did the scientific literature review and extracted the data. KDR, KP, and JHB did the statistical analyses, reviewed the results, wrote the manuscript, and revised it following critical review by all authors. All authors take responsibility for the integrity and accuracy of the data analysis and the decision to submit this paper for publication. All authors read and approved the final manuscript.

## Pre-publication history

The pre-publication history for this paper can be accessed here:

http://www.biomedcentral.com/1471-2458/13/941/prepub

## Supplementary Material

Additional file 1 Table A-E and Figure A related to operationalisation of the covariates, description of missing variables, multiple imputation analyses, creation of the propensity score, and matched propensity score analyses. Click here for file
