# Supplementary material for: High school dropout and long-term sickness and disability in young adulthood: a prospective propensity score stratified cohort study (the Young-HUNT study)
Source: BMC Public Health. 2013 Oct 9;13:941. doi: 10.1186/1471-2458-13-941 (PMC4124891; doi:10.1186/1471-2458-13-941)
Supplement: Additional file 1 — Table A-E and Figure A related to operationalisation of the covariates, description of missing variables, multiple imputation analyses, creation of the propensity score, and matched propensity score analyses. [file 1471-2458-13-941-S1.doc]

**Appendix**

**Table A**. Operationalisation of the covariates

| Chronic somatic disease | Self-reported asthma, diabetes, migraine, or epilepsy diagnosed by a doctor or any other illness that lasted longer than 3 months. Dichotomized into “yes” or “no”. |
| --- | --- |
| Symptom load | The sum of self-reported presence (dichotomized into “never/seldom” and “sometimes/often”) of eight symptoms (headache, neck or shoulder pain, joint or muscle pain, stomach pain, nausea, constipation, diarrhea, heart palpitations) during the last 12 months. Range 0 (no symptoms) to 8 (8 symptoms) |
| Psychological distress | A mean score based on self-reported symptoms of anxiety and depression measured by SCL-5 scale score – a validated 4-integer 5 item short version of the original SCL-90*. Continuous variable with range 1 (low) to 4 (high psychological distress). (*Hopkins symptom checklist) |
| Concentration problems | Self-reported difficulties concentrating during class and dichotomized into “never/sometimes” versus “often/ very often”. |
| Self-rated health | Self-rated health using the question “How is your health at the moment?” and dichotomized into “good/very good” versus “poor/not so good”. |
| Insomnia | Difficulties falling asleep in the last month and dichotomized into “never/sometimes” versus “often/ almost every night” |
| BMI | Overweight corresponded with the adult BMI from 25 to 30 and obesity a BMI of 30 and more. |
| Smoking | Smokers are those who answered “Yes” to the question about ever smoking combined with answering “Yes, I smoke daily” or “Yes, I smoke occasionally” to the question “Do you smoke now?” Non- smokers are those who had never smoked cigarettes or had stopped smoking. |
| Physical activity | Self-reported activity about if they exercise until they get out of breath or sweat outside school hours was dichotomized into inactive (less than every 14^th^ day) or active (more than every 14^th^ day). |
| Self-esteem | A mean score based on a short version of the Rosenberg Self-Esteem Scale consisting of 4-integer 4 item version. Range 1 (low) to 4 (high self-esteem). |
| Subjective well-being | A mean score based on 3 questions with each a 7-point scale from very satisfied to very dissatisfied. Range 1 (high) to 7 (low subjective well-being). |
| Loneliness | The question “Do you feel lonely?” with a 5-point scale from “very often” to “very seldom or never”. |
| Family living situation | Living in a traditional family (with both the biological mother and father) or not. |
| Reading and writing difficulties | Defined as responding “yes” to the question “Do you currently receive help for reading or writing problems?” or reporting major problems with either reading or writing the last 12 months. |
| Being bullied | Response on the statement “You are teased/harassed by other students” with a 4-point scale from “never” to “very often”. |
| Educational aspirations | Based on the question “What type of plans do you have regarding continued studies?” categorized into 3 groups (“none/do not know”, “high school” and “higher education”) |
| Disease-related absence from school | Reporting more than 2 weeks absence from school due to illness during the last 12 months. |
| Academic problems | A mean score based on self-reported problems related to academic achievement based on 5 questions with a 4-point scale. Range 1 (never) to 4 (very often). |
| School-related dissatisfaction | A mean score based on self-reported problems related to dissatisfaction at school based on 4 questions with a 4-point scale. Range 1 (never) to 5 (very often). |
| School-related conduct | A mean score based on self-reported school-related conduct problems based on 4 questions with a 4-point scale. Range 1 (never) to 5 (very often). |

**APPENDIX**

**Table B.** Number of missing variables (% in parenthesis) in the dataset (N=8816) for the whole population and stratified by the variable “school dropout”.

|  | Total  n=8816 | School dropouts  n=1474 | School completers  n=7342 |
| --- | --- | --- | --- |
| *Demographics* |  |  |  |
| Age, mean | 0 (0) | 0 (0) | 0 (0) |
| Male | 0 (0) | 0 (0) | 0 (0) |
| *Maternal education level* | 39 (0.4) | 20 (1.4) | 19 (0.3) |
| *Health* |  |  |  |
| 1 or more somatic disease | 0 (0) | 0 (0) | 0 (0) |
| Symptom load, mean | 314 (3.6) | 80 (5.4) | 234 (3.2) |
| High psychological distress | 169 (1.9) | 68 (4.6) | 101 (1.4) |
| Concentration problems | 180 (2.0) | 69 (4.6) | 111 (1.5) |
| Insomnia | 132 (1.5) | 52 (3.5) | 80 (1.1) |
| Poor self-rated health | 148 (1.7) | 35 (2.4) | 113 (1.5) |
| *Health behavior* |  |  |  |
| BMI | 545 (6.2) | 168 (11.4) | 377 (5.1) |
| Smoking | 208 (2.4) | 50 (3.4) | 158 (2.2) |
| No physical activity | 116 (1.3) | 37 (2.6) | 79 (1.1) |
| *Psychosocial factors* |  |  |  |
| Self-esteem | 219 (2.5) | 81 (5.5) | 138 (1.9) |
| Subjective well-being | 133 (1.5) | 50 (3.4) | 83 (1.1) |
| Loneliness | 137 (1.5) | 54 (3.7) | 83 (1.1) |
| Traditional family | 128 (1.5) | 34 (2.3) | 94 (1.3) |
| *School-related factors* |  |  |  |
| Reading and writing difficulties | 307 (3.5) | 103 (7.0) | 202 (2.8) |
| Being bullied | 224 (2.5) | 83 (5.6) | 141 (1.9) |
| Disease-related school absence | 522 (5.9) | 180 (12.2) | 342 (4.7) |
| Aspiration for higher education | 306 (3.5) | 75 (5.1) | 231 (3.1) |
| Academic problems | 360 (4.1) | 117 (7.9) | 243 (3.3) |
| School-related dissatisfaction | 454 (5.1) | 141 (9.6) | 313 (4.3) |
| School-related conduct | 325 (3.7) | 102 (7.0) | 223 (3.0) |

**APPENDIX**

**Table C.** Odds ratio of receiving long-term medical benefits between age 24 to 29 years for high school dropouts compared to school completers in the whole population for complete cases analysis (logistic regression models, N=6607) and analysis based on multiple imputations (logistic regression models, N=8805).

|  | Model 0 | Model 1 | Model 2 | Model 3 | Model 4 | Model 5 |
| --- | --- | --- | --- | --- | --- | --- |
| **dropout *versus* completion (ref.)** | |  |  |  |  |  |
| **Complete cases** |  |  |  |  |  |  |
| Odds ratio | 3.93  (3.29 to 4.70) | 3.54  (2.95 to 4.25) | 3.36  (2.80 to 4.05) | 3.21  (2.66 to 3.87) | 3.08  (2.55 to 3.73) | 2.96  (2.44 to 3.59) |
| **Multiple imputation** |  |  |  |  |  |  |
| Odds ratio | 3.88  (3.37 to 4.47) | 3.57  (3.09 to 4.13) | 3.35  (2.90 to 3.88) | 3.12  (2.67 to 3.65) | 2.94  (2.52 to 3.45) | 2.74  (2.32 to 3.22) |

*Estimated risk difference in the 6-year risk to receive long-term medical benefits with the covariates at their mean.

Model 0: adjusted for sex, age and follow-up time

Model 1: model 0 +adjusted for maternal education level.

Model 2: model 1 + adjusted for somatic disease, symptom load, psychological distress, concentration problems, insomnia, and self-rated health.

Model 3: model 2 + adjusted for overweight, smoking, and physical activity.

Model 4: model 3 + adjusted for self-esteem, subjective well-being, loneliness, and family living situation.

Model 5: model 4 + adjusted for reading and writing difficulties, bullying, disease-related school absence, educational aspirations, school dissatisfaction, and school-related conduct.

**Multiple imputation procedure:**

**Assumption: missing at random**

Basically, the fraction of missing data for each variable was low and varied from 0% to 6.2%.

83% of the participants with missing data had missing data for only 1 or 2 variables.

The variable with most missing data was “BMI”, which was based on the measurement of height and weight by nurses. 388 of the participants with missing information on BMI (71%) had a full completed questionnaire (no other missing data) . It is unlikely that they could not meet up or be measured because of their BMI itself. It could be possible that they were less willing to meet up because of a psychological reluctancy, but we included several psychological measurements in the dataset that can predict the probability of missingness (psychological distress, self-esteem, subjective well-being) and the incomplete variable. It could also be possible that a group of individuals are less dutyful, and in that case the variable “school dropout” might (as a proxy) predict the probability of missingness and the incomplete variable. Finally, some participants were maybe not able to meet up, completely at random: e.g. had to catch the buss, had to be in another class/examination, and so on.

**Variable selection and imputation model**

*Multiple imputation with chained equations with*

- *Imputing binary variables with logistic regression (logit):*

Symptom load, psychological distress, insomnia, concentration problems, self-reported health, smoking, physical activity, self-esteem, subjective well-being, lonileness, family living situation, reading and writing difficulties, being bullied, school-related dissatisfaction, school-related conduct problems, disease-related school absence

- *Imputing ordered categorical variables with ordinal logistic regression (ologit):*

Maternal education level, educational aspiration, BMI

- *Using outcome, auxiliary outcomes and other not imputed variables:*

Medical benefits between age 24-29, unemployment between age 24-29, social assistance between age 24-29, age, sex, somatic disease, follow-up time, linkage between siblings

- *Variable of complete case analysis not included in multiple imputation:*

Variable “academic problems” was omitted because of too much collinearity with “concentration problems”.

Note 1: No interactions were included because the complete case analyses showed no statistical significant interactions.

Note 2: Because of practical difficulties, all imputed variables were included as either binary or ordinal categorical variables.

**Number of imputations and estimates**: The 20 created datasets were individually but identically analyzed. The estimates were combined to obtain overall estimates and confidence intervals.

**APPENDIX**

**Table D.** Log odds ratios of high school dropout and standard errors for the variables included in the propensity analysis. (N=6612)

|  |  |  | 95% confidence interval | |  |
| --- | --- | --- | --- | --- | --- |
|  | Coefficient | Standard error | Lower | Upper | P-value |
| Sex | 0.573 | 0.086 | 0.404 | 0.742 | <0.0001 |
| Age | -0.136 | 0.024 | -0.183 | -0.089 | <0.0001 |
| Maternal education level |  |  |  |  |  |
| Intermediate | -0.474 | 0.084 | -0.639 | -0.310 | <0.0001 |
| Tertiary | -1.114 | 0.123 | -1.356 | -0.874 | <0.0001 |
| Chronic somatic disease | 0.104 | 0.092 | -0.077 | 0.280 | 0.26 |
| Symptom load | 0.028 | 0.029 | -0.028 | 0.085 | 0.32 |
| Psychological distress | -0.119 | 0.103 | -0.321 | 0.083 | 0.25 |
| Concentration problems | -0.182 | 0.109 | -0.396 | 0.032 | 0.09 |
| Insomnia | 0.202 | 0.123 | -0.403 | 0.445 | 0.10 |
| Self-rated health | 0.035 | 0.124 | -0.202 | 0.271 | 0.77 |
| BMI |  |  |  |  |  |
| Overweight | 0.291 | 0.102 | 0.090 | 0.491 | 0.005 |
| Obese | 0.621 | 0.188 | 0.253 | 0.989 | 0.001 |
| Smoking | 0.490 | 0.092 | 0.310 | 0.670 | <0.0001 |
| Physical activity | 0.418 | 0.106 | 0.209 | 0.626 | <0.0001 |
| Self-esteem | -0.102 | 0.090 | -0.278 | 0.075 | 0.26 |
| Subjective wellbeing | -0.076 | 0.057 | -0.188 | 0.037 | 0.18 |
| Loneliness | -0.043 | 0.050 | -0.140 | 0.054 | 0.38 |
| Family living situation | -0.645 | 0.081 | -0.805 | -0.486 | <0.0001 |
| Reading and writing difficulties | 0.697 | 0.117 | 0.466 | 0.928 | <0.0001 |
| Being bullied | 0.050 | 0.081 | -0.110 | 0.209 | 0.54 |
| Educational aspirations | -0.090 | 0.047 | -0.183 | 0.003 | 0.06 |
| Disease-related absence from school | 0.600 | 0.152 | 0.301 | 0.898 | <0.0001 |
| Academic problems | 1.356 | 0.139 | 1.082 | 1.630 | <0.0001 |
| School-related dissatisfaction | -0.127 | 0.089 | -0.302 | 0.048 | 0.16 |
| School-related conduct | -0.073 | 0.099 | -0.267 | 0.120 | 0.45 |
| Constant | -0.967 | 0.551 | -2.047 | 0.112 | 0.08 |

**APPENDIX**

**Figure A.** Comparison of box plots of propensity score for likelihood of dropping out of school.

**APPENDIX**

**Table E.** Average effects of treatment on treated (ATT) with and without restriction of the region of common support, including bootstrapped propensity scores and standard error (SE) with 100 replications of the ATT.

| Matching method | School dropouts, n | School completers, n | ATT | SE | 95% CI^a^ for ATT |
| --- | --- | --- | --- | --- | --- |
| Region of common support: propensity score 0.0163-0.9392 (n=6550) | | | | | |
| Radius (0.1) | 910 | 5640 | 0.165 | 0.015 | 0.136-0.194 |
| Region of common support not imposed: propensity score 0.0081-0.9414 (n=6612) | | | | | |
| Radius (0.1) | 910 | 5702 | 0.165 | 0.015 | 0.136-0.194 |

^a^Confidence interval
